# How conductance distributions are shaped by activity-dependent regulation rules

**DOI:** 10.1186/1471-2202-14-S1-P258

**Published:** 2013-07-08

**Authors:** Timothy O'Leary, Alex H Williams, Jonathan S Caplan, Eve Marder

**Affiliations:** 1 Volen Center for Complex Systems, Brandeis University, Waltham, MA 02454, USA

## 

Neurons co-express a multitude of different ion channels that determine the electrophysiological behaviour of the cell. Recent experimental observations have revealed that the expression of different ion channels varies dramatically within neurons of a defined type, even when these neurons exhibit stereotyped electrical properties [1-5]. This variability has a structure visible in the correlations between different ion channel expression levels. Nonetheless,, the underlying mechanism that determines these correlations is unknown, nor is understood how these correlations can coexist with activity-dependent regulation mechanisms that are also known to exist in neurons [6]. We show that these observations are logical consequences of relatively simple control mechanisms that couple the expression levels of individual conductances to a cell-intrinsic readout of activity [7]. Crucially, these correlations are not visible when conductance space is searched to find combinations of conductances that give target behaviour. Therefore, activity-dependent regulation mechanisms constrain the solution space of potential combinations of membrane conductances to a characteristic subset. Furthermore, we show how the shape of the conductance distribution of a population of neurons is determined by the relative rates of expression of different conductances. Finally, degeneracy in the function of multiple ion channels means that regulation mechanisms can have widely-variable parameters yet remain stable; this is exemplified by tolerance to 'anti-homeostatic' regulation of a subset of conductances.
